# Investigation of the Impact of miRNA-7151 and a Mutation in Its Target Gene lncRNA *KCNQ1OT1* on the Pathogenesis of Preeclampsia

**DOI:** 10.3390/biomedicines13081813

**Published:** 2025-07-24

**Authors:** Wuqian Wang, Xiaojia Wu, Jianmei Gu, Luan Chen, Weihua Zhang, Xiaofang Sun, Shengying Qin, Ping Tang

**Affiliations:** 1 Guangdong Provincial Key Laboratory of Major Obstetric Diseases, Guangdong-Hong Kong-Macao Greater Bay Area Higher Education Joint Laboratory of Maternal-Fetal Medicine, Department of Obstetrics and Gynecology, Guangdong Provincial Clinical Research Center for Obstetrics and Gynecology, The Third Affiliated Hospital, Guangzhou Medical University, Guangzhou 510180, Chinaxiaofangsun@hotmail.com (X.S.); 2 Jiaxing Maternity and Children Health Care Hospital, Affiliated Women and Children Hospital Jiaxing University, Jiaxing 314051, China; lingzhuwei@126.com (X.W.); gjmcindy@163.com (J.G.); 18967391875@163.com (W.Z.); 3 Key Laboratory for the Genetics of Developmental and Neuropsychiatric Disorders, Bio-X Institutes, Ministry of Education, Shanghai Jiao Tong University, Shanghai 200030, China; clmelody@163.com

**Keywords:** preeclampsia, miRNAs, exosomes, biomarkers, placenta

## Abstract

**Background**: Preeclampsia (PE) is a pregnancy-specific disease and hypertensive disorder with a multifactorial pathogenesis involving complex molecular regulatory networks. Recent studies highlight the critical role of non-coding RNAs, particularly miRNAs and lncRNAs, in PE development. This study investigates the molecular interaction between miR-7151-5p and the lncRNA *KCNQ1OT1* and their functional contributions to PE pathogenesis. **Methods**: An integrative approach combining RNAhybrid-based bioinformatics, dual-luciferase reporter assays, qRT-PCR, Transwell migration and invasion assays, and RNA sequencing was employed to characterize the binding between miR-7151-5p and *KCNQ1OT1* and assess their influence on trophoblast cell function and gene expression. **Results**: A bioinformatic analysis predicted a stable binding site between miR-7151-5p and *KCNQ1OT1* (minimum free energy: –37.3 kcal/mol). The dual-luciferase reporter assay demonstrated that miR-7151-5p directly targets *KCNQ1OT1*, leading to suppressed transcriptional activity. In HTR8/SVneo cells, miR-7151-5p overexpression significantly downregulated both *KCNQ1OT1* and Notch1 mRNA, whereas its inhibition showed no significant changes, suggesting additional regulatory mechanisms of Notch1 expression. Transwell assays indicated that miR-7151-5p overexpression suppressed trophoblast cell migration and invasion, whereas its inhibition enhanced these cellular behaviors. RNA-seq analysis further revealed that miR-7151-5p overexpression altered key signaling pathways, notably the TGF-β pathway, and significantly modulates PE-associated genes, including *PLAC1*, *ANGPTL6*, *HIRA*, *GLA*, *HSF1*, and *BAG6*. **Conclusions**: The regulatory effect of miR-7151-5p on *KCNQ1OT1*, along with its influence on trophoblast cell dynamics via Notch1 and TGF-β signaling pathways, highlights its role in PE pathogenesis and supports its potential as a biomarker in early PE screening.

## 1. Introduction

Preeclampsia (PE) is a gestational hypertensive disorder that typically emerges after 20 weeks, accompanied by proteinuria, and represents a major risk to the health of both the mother and fetus [1]. In severe cases, it can lead to maternal and fetal mortality. Affecting 3–8% of pregnancies globally, PE remains a significant contributor of maternal and perinatal complications [1]. Although its precise etiology remains unclear, placental dysfunction is widely recognized as a pivotal factor in its pathogenesis [2].

Exosomes play crucial roles in pregnancy-related processes [3]. Significant alterations in peripheral blood exosomal miRNA profiles among individuals with PE have been reported, highlighting their possible contribution to the condition’s progression [4,5,6,7]. These exosomal miRNAs have been implicated in impaired trophoblast invasion, the promotion of endothelial dysfunction, and impaired angiogenesis—key features of PE [8]. These results suggest that exosomal miRNAs hold potential as early screening markers and therapeutic targets for PE. In recent years, there has been rising recognition of the significant role played by non-coding RNAs (ncRNAs) in PE progression [9]. Dysregulated miRNA expression is frequently detected in PE patients and has been shown to influence trophoblast activity and placental development [2]. For instance, miR-210 is upregulated in PE and impairs trophoblast invasion by regulating the MAPK signaling pathway, thereby affecting trophoblast function and placental development; it is also regarded as an important serum biomarker for early PE prediction [10]. Similarly, miR-181 shows significant overexpression in PE [11]. As another example, miR-362-3p negatively regulates trophoblast proliferation, migration, and invasion by targeting *Pax3* [12].

Our previous study identified the significant upregulation of miRNA-7151 in the exosomes of patients with PE, with lncRNA *KCNQ1OT1* suggested as a potential downstream regulatory target. However, the precise function of miR-7151-5p in PE development has yet to be fully understood [13]. lncRNAs are associated with the control of vital cellular activities like proliferation, differentiation, and programmed cell death [14]. Among them, *KCNQ1OT1* has emerged as an important regulatory molecule implicated in various diseases [15]. *KCNQ1OT1* has been reported to control cellular processes such as proliferation, migration, and invasion by modulating miRNA levels [15]. Notably, *KCNQ1OT1* has been recognized as a diagnostic and therapeutic biomarker of several diseases, including rectal cancer, cerebral ischemia–reperfusion injury, and diabetic nephropathy, where it is highly expressed in affected tissues [16,17,18].

In the context of PE, one study reported that *KCNQ1OT1* negatively regulates miR-146a-3p, thereby activating the CXCL12/CXCR4 signaling pathway potentially contributing to disease pathogenesis [19]. Moreover, *KCNQ1OT1* expression is transcriptionally regulated by multiple transcription factors, including β-catenin and c-Myc [20]. β-Catenin, for example, can bind directly to the *KCNQ1OT1* promoter and enhance its transcription, influencing downstream genes [21]. Despite these insights, the regulatory mechanism of *KCNQ1OT1* in remains incompletely understood [19].

Recent studies have increasingly highlighted the importance of miRNA–lncRNA interactions as key regulators in the development of PE, primarily by modulating trophoblast function, oxidative stress, ferroptosis, and placental development [2,22,23,24]. *KCNQ1OT1* has emerged as a well-characterized lncRNA that acts as a competing endogenous RNA (ceRNA) in various diseases, including PE. It regulates ferroptosis-related genes such as *SLC7A11* and *GPX4*, thereby influencing redox homeostasis and angiogenesis in the placenta [22,23]. Transcriptomic network analysis supports *KCNQ1OT1* as a hub lncRNA within lncRNA–mRNA co-expression modules [24]. Based on this evidence, investigating the miR-7151-5p–*KCNQ1OT1* axis offers novel insights into early-onset PE and provides a compelling rationale for further mechanistic exploration.

Our previous study provided preliminary evidence of a regulatory relationship between miR-7151-5p and *KCNQ1OT1* in PE, suggesting their involvement in gene expression regulation associated with the disease [13]. However, it remains unclear whether miR-7151-5p directly binds to *KCNQ1OT1*, whether this interaction exerts functional effects on gene regulation in early-onset PE, and which disease-related signaling pathways are involved. The objective of this research is to elucidate the interaction between miR-7151-5p and the lncRNA *KCNQ1OT1*, characterize their biological functions in the context of PE, and uncover the signaling mechanisms involved. This work intends to advance current knowledge of PE pathophysiology and offer a foundation for future early screening strategies.

## 2. Materials and Methods

### 2.1. Human Chorionic Trophoblast Cell Culture

The HTR-8/SVneo cell line, derived from human extravillous trophoblast (hereafter referred to as HTR-8) was obtained from Shanghai Yizefeng Biotechnology Co., Ltd. (Shanghai, China). For culture, cells were cultured in RPMI-1640 medium (Gibco, Billings, MT, USA), which was supplemented with 10% fetal bovine serum (FBS), 100 U/mL penicillin, and 100 μg/mL streptomycin [25]. When cells reached 80–90% confluence, they were detached using 0.25% trypsin-EDTA [25].

### 2.2. Bioinformatics Prediction of the Binding Sites Between miR-7151-5p and KCNQ1OT1

RNAhybrid software(version 2.1.2) was utilized to conduct a bioinformatic analysis to identify potential binding sites between miR-7151-5p and the lncRNA *KCNQ1OT1* [26]. The mature sequence of miR-7151-5p was obtained from the miRBase database(version 22.0) [27], and the full nucleotide sequence of *KCNQ1OT1* was accessed via the Ensembl database(version 110) [28]. Base pairing predictions were performed using the RNAhybrid online tool [26], which calculated the minimum free energy (MFE) of hybridization to assess binding potential.

Binding predictions were validated based on two criteria: a free energy threshold of ΔG ≤ −20 kcal/mol and strong base pairing within the miRNA seed region [26]. The candidate binding sites identified by RNAhybrid were subsequently selected for functional validation using dual-luciferase reporter assays [26].

### 2.3. Dual-Luciferase Reporter Assay

To verify the interaction between miR-7151-5p and the predicted sites on lncRNA *KCNQ1OT1*, a dual-luciferase reporter assay was performed [29]. The WT fragment of *KCNQ1OT1* with the putative miR-7151-5p-binding site was subcloned into the pMIR-REPORT luciferase vector (Ambion, Austin, TX, USA) [29]. To assess binding specificity, a mutant (MUT) construct with point substitutions at the target sites was generated in parallel [29]. HTR-8 cells in 24-well plates were co-transfected with 500 ng of either *KCNQ1OT1*-WT or MUT luciferase constructs and 50 nM miR-7151-5p mimic or NC mimic (Lipofectamine 3000, Invitrogen, Carlsbad, CA, USA) [30]. The internal control was a Renilla luciferase plasmid (pRL-TK, Promega, Madison, WI, USA) [30]. Firefly/Renilla ratios were calculated with a GloMax luminometer [31].

### 2.4. miR-7151-5p Overexpression and Inhibition Experiments

To explore the functional effects of miR-7151-5p in the trophoblast cells, miR-7151-5p mimics and inhibitors (2′-O-Methyl modified, chemically synthesized) were purchased from Platinum Biosciences Co., Ltd. (Shanghai, China) [32]. When HTR-8/SVneo cells reached approximately 80% confluence, they were trypsinized with 0.25% trypsin-EDTA, reseeded at a 30% density in 24-well plates, and incubated overnight for attachment [33]. The following day, when the cells reached ~60% confluence, which was widely recommended to ensure optimal cell health and transfection efficiency, while minimizing cytotoxicity and overgrowth during post-transfection incubation periods [34], Lipofectamine 3000 (Invitrogen, Carlsbad, CA, USA) was used for miRNA transfection [35]. The study included four experimental groups, each performed in triplicate: blank control, fluorescent NC control, miR-7151-5p mimic, and miR-7151-5p inhibitor [35].

For each replicate, two 1.5 mL centrifuge tubes were prepared, each containing 50 μL of Opti-MEM serum-free medium (Gibco, Billings, MT, USA). In one tube, 2 μL of Lipofectamine 3000 was added; in the other, the mimic or inhibitor was added to 100 nM, consistent with concentrations commonly used in functional miRNA assays [36].

The culture medium in each well was refreshed with complete medium, followed by the dropwise addition of 100 μL of the transfection complex [36]. The plates were gently agitated to ensure uniform distribution and then incubated for 6 h [36]. Cells were maintained for an additional 48 h before proceeding with subsequent experiments [36].

### 2.5. Total RNA Extraction and qRT-PCR

RNA was isolated from HTR-8/SVneo cells using TRIzol reagent (Invitrogen, Carlsbad, CA, USA) [37]. The RNA concentration and purity were measured using a NanoDrop (Thermo Fisher, Waltham, MA, USA) [37]. cDNA was generated using the PrimeScript™ RT Kit (Takara, Osaka, Japan) [37]. QRT-PCR was performed on a Bio-Rad CFX96 system, with TB Green^®^ Premix Ex Taq™ II (Takara, Osaka, Japan) [37]. GAPDH was used as the internal reference, and relative expression was calculated based on the 2^−ΔΔCt^ method [37]. Primers were as follows: KCNQ1OT1, forward: AGGGTGACAGTGTTTCATAGGCT, reverse: GAGGCACATTCATTCGTTGGT; NOTCH1, forward: AGAGGCGTGGCAGACT, reverse: TGTACTCCGTCAGCGTGAG; GAPDH, forward: GAAAGCCTGCCGGTGACTAA, reverse: TTCCCGTTCTCAGCCTTGAC.

### 2.6. Assessment of the Effects of Overexpression or Knockdown of Candidate miRNAs on Migration and Invasion of Chorionic Trophoblast Cells

HTR-8/SVneo cells were transfected with either miR-7151-5p mimics or inhibitors [35]. Forty-eight hours later, the cells were subjected to Transwell experiments to assess their migratory and invasive behavior [35]. These experiments were assessed with 24-well Transwell chambers (Corning, Corning, NY, USA, Cat#3422) [38]. HTR-8/SVneo cells (5 × 10^4^) in serum-free RPMI-1640 were added to the upper chamber, while 10% FBS-containing medium was placed in the lower chamber [38]. After 24 h, non-migrated cells were removed; migrated cells were fixed, stained with crystal violet, and counted in five random 200× fields [38]. The experiment was set up with three replicate wells and repeated three times independently [38]. For the invasion experiment, the difference was that the upper chamber membrane was pre-coated with 1:8-diluted Matrigel gel (Corning, Corning, NY, USA, Cat#356234), and the other procedure was the same as that of the migration experiment [38].

Images were analyzed and cells were counted using ImageJ software (version 1.51) (NIH, Bethesda, MD, USA) [39]. Counts were averaged from five random fields per well [39]. Data were shown as the mean ± SD [39]. One-way ANOVA was used for analysis in GraphPad Prism 9.0, with *p* < 0.05 deemed significant [39].

### 2.7. RNA Extraction and Transcriptome Sequencing

In order to study the influence of miR-7151-5p overexpression and inhibition on global gene expression, transcriptome sequencing was conducted on HTR-8/SVneo cells transfected with miR-7151-5p mimics or inhibitors [34].

Total RNA was isolated 48 h after transfection using TRIzol reagent (Invitrogen, Carlsbad, CA, USA) [37]. The RNA quality and concentration were measured with a NanoDrop, and integrity was checked using an Agilent 2100 Bioanalyzer [37]. Only samples meeting quality standards were used for subsequent sequencing [37]. RNA libraries were prepared from each sample, and sequencing was performed on an Illumina NovaSeq 6000, with PE150 (Illumina, San Diego, CA, USA) [37]. Quality control procedures were applied prior to library preparation and sequencing [37].

### 2.8. Data Preprocessing and Differentially Expressed Gene Analysis

The raw sequencing data quality was evaluated using FastQC [40]. Adapter sequences along with low-quality reads were trimmed using Trimmomatic (v0.39) [40]. Cleaned reads were mapped to the human reference genome (hg 19) with Hisat2 (v2.1.0), followed by transcript assembly and gene expression quantification with StringTie (v2.1.4) [41]. DESeq2 (v1.28.1) was used to identify significantly DEGs, defined by a |log2 fold change| > 1 and *p* < 0.05 [42].

### 2.9. Functional Annotation and Pathway Enrichment Analysis

DEG functional enrichment was analyzed using Kyoto Encyclopedia of Genes and Genomes (KEGG) and Gene Ontology (GO) databases [43] to identify signaling pathways potentially regulated by miR-7151-5p. The clusterProfiler R package (version 4.3.1) was used to perform enrichment analysis, applying the Benjamini–Hochberg method to adjust *p*-values [44]. *p*-values below 0.05 were used [44].

## 3. Results

### 3.1. Bioinformatics Prediction of the Binding Sites Between miR-7151-5p and KCNQ1OT1

**Figure 1:**
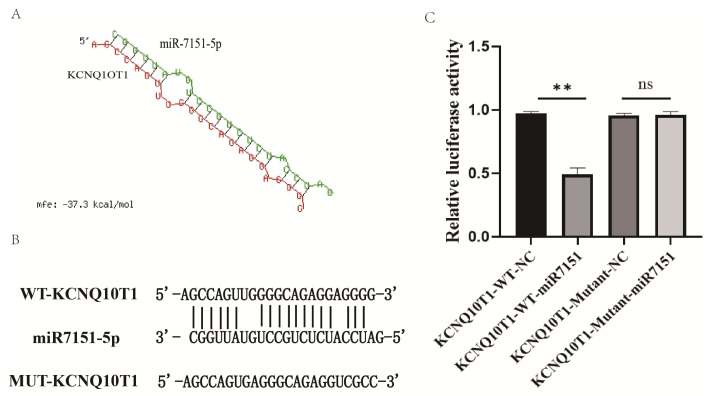
Validation of the interaction between miR-7151-5p and *KCNQ1OT1* through bioinformatic prediction and dual-luciferase reporter assay. (**A**) Prediction of binding sites between miR-7151-5p and the target gene *KCNQ1OT1* using RNAhybrid software; (**B**) sequence of the predicted binding site; (**C**) dual-luciferase reporter assay validating the interaction between miR-7151-5p and KCNQ1OT1. WT: wild-type sequence; Mutant: mutated sequence; The psiCHECK2 plasmid was used for vector construction. Significance: *p* < 0.01 (**), not significant (ns).

To explore whether miR-7151-5p directly targets the IncRNA *KCNQ1OT1*, the RNAhybrid tool was used [26]. The analysis revealed a stable and complementary interaction between miR-7151-5p and the wild-type (WT) sequence of *KCNQ1OT1*, with an MFE of −37.3 kcal/mol, which indicates a high binding affinity (Figure 1A). The predicted binding region and detailed base-pairing interactions are illustrated in Figure 1B.

### 3.2. Dual-Luciferase Reporter Assay Validates Direct Binding Between miR-7151-5p and KCNQ1OT1

The *KCNQ1OT1* 3′UTR fragment containing the putative miR-7151-5p-binding site was inserted into the psiCHECK2 vector. Both wild-type and mutant constructs were co-transfected with miR-7151-5p mimics or the negative control into HEK293T cells. As illustrated in Figure 1C, luciferase activity was inhibited in the KCNQ1OT1-WT co-transfected with miR-7151-5p group compared to the KCNQ1OT1-WT + NC group (*p* < 0.01), indicating that miR-7151-5p can directly bind to and suppresses *KCNQ1OT1* expression. Conversely, the mutant construct showed no significant change in luciferase activity (KCNQ1OT1-Mutant) following co-transfection with miR-7151-5p mimics (*p* > 0.05), suggesting that the introduced mutation disrupted the miRNA-binding site.

### 3.3. miR-7151-5p Overexpression and Inhibition Experiments

**Figure 2:**
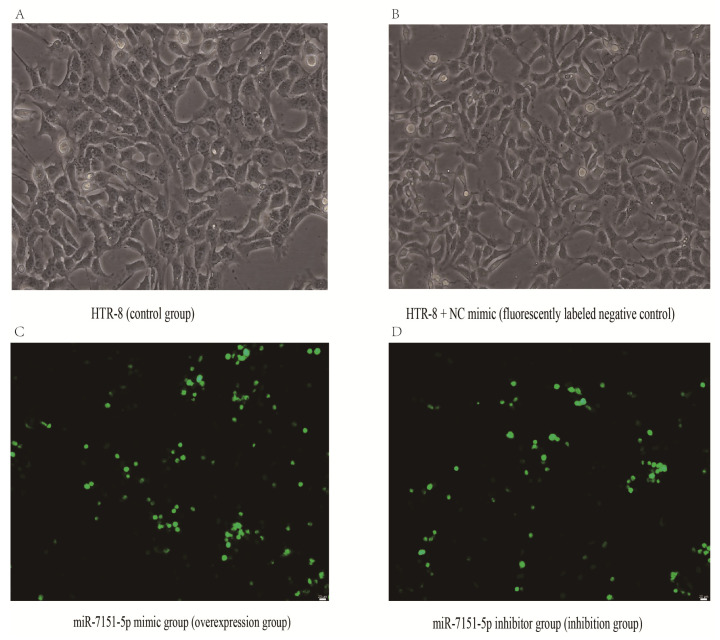
Experimental groups and transfection strategy using the chorionic trophoblast cell line HTR-8/SVneo. To investigate the functional role of miR-7151-5p in trophoblast cells, four experimental groups were established and transfected accordingly: (**A**) HTR-8 (control group): untreated blank control group, used to assess baseline cell behavior without any transfection reagents. (**B**) HTR-8 + NC mimic (fluorescently labeled negative control): cells were transfected with a non-targeting miRNA mimic labeled with a fluorescent dye. (**C**) miR-7151-5p mimic group (overexpression group): cells were transfected with chemically synthesized miR-7151-5p mimics to artificially increase its intracellular levels. (**D**) miR-7151-5p inhibitor group (inhibition group): cells were transfected with miR-7151-5p inhibitors to reduce endogenous miR-7151-5p activity. All groups were analyzed 48 h post-transfection for subsequent phenotypic and molecular assays.

To explore the regulatory effect of miR-7151-5p on its predicted target gene *KCNQ1OT1* and the downstream gene *Notch1* in human chorionic trophoblast cells (HTR8/SVneo), four experimental groups were established: miR-7151-5p overexpression group (mimic), miR-7151-5p inhibition group (inhibitor), negative control group (NC), and untreated control, as illustrated in Figure 2. The control group (HTR-8) consisted of untreated cells to provide a baseline for the comparison. A negative control group (HTR-8 + NC mimic) was transfected with a fluorescently labeled non-targeting miRNA mimic to control for nonspecific transfection effects. To induce overexpression, the miR-7151-5p mimic group was transfected with synthetic miR-7151-5p molecules, whereas the miR-7151-5p inhibitor group received specific inhibitors to downregulate endogenous miR-7151-5p activity. All groups were analyzed 48 h after transfection for downstream molecular and phenotypic assays. qRT-PCR was used to quantify the mRNA levels of miR-7151-5p and its target genes, with each experiment conducted in triplicate.

**Figure 3:**
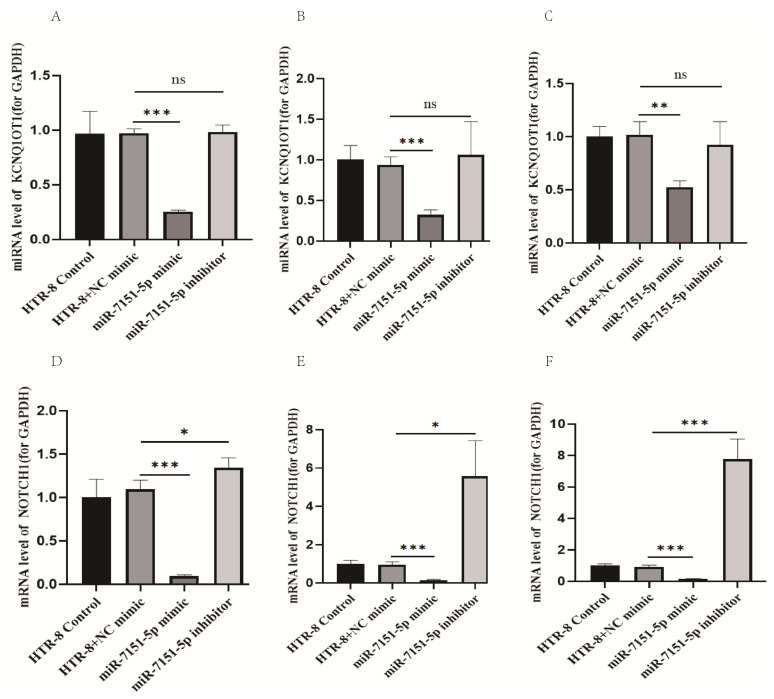
Regulatory relationship between miRNAs and candidate target genes. This figure illustrates the expression changes of KCNQ1OT1, a predicted direct target of miR-7151-5p, and its downstream effector Notch1, a known regulator of trophoblast function and placental development. Experimental groups included miR-7151-5p overexpression (mimic) and knockdown (inhibitor), along with their respective negative controls. (**A**–**C**) Expression levels of the candidate target gene *KCNQ1OT1* regulated by miR-7151-5p. (**D**–**F**) Expression levels of the downstream target gene Notch1 of KCNQ1OT1, regulated by miR-7151-5p. Significance: *p* < 0.001 (***), *p* < 0.01 (**), *p* < 0.05 (*), not significant (ns).

Figure 3A–C demonstrate that *KCNQ1OT1* expression was significantly decreased in the miR-7151-5p mimic group (*p* < 0.001), whereas the inhibitor group showed no notable difference (*p* > 0.05), implying that miR-7151-5p exerts a suppressive effect on *KCNQ1OT1* expression. Likewise, Notch1 expression was significantly downregulated in the miR-7151-5p mimic group (Figure 3D–F, *p* < 0.001), while no notable difference was observed following miR-7151-5p inhibition (*p* > 0.05). These results imply that miR-7151-5p may regulate Notch1 expression either directly or through alternative pathways, independent of *KCNQ1OT1*.

The miR-7151-5p inhibitor group showed no significant change in target gene expression, possibly due to compensatory mechanisms or low baseline miR-7151-5p levels.

### 3.4. Assessment of the Effects of Overexpression or Knockdown of Candidate miRNAs on Migration and Invasion of Chorionic Trophoblast Cells

**Figure 4:**
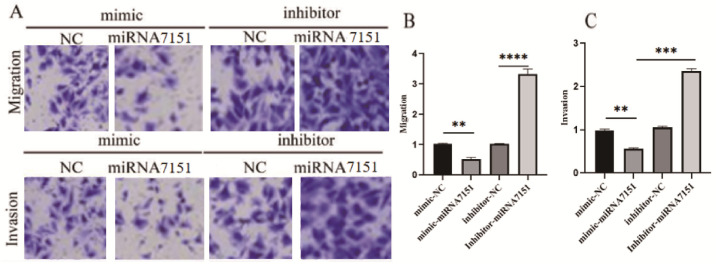
MiR-7151-5p regulates migration and invasion in chorionic trophoblast cells. (**A**) Representative images from Transwell migration (upper) and invasion (lower) assays of HTR-8/SVneo cells transfected with miR-7151-5p mimic or inhibitor. Cells were stained with crystal violet after 48 h post-transfection. (**B**) Quantification of migrated cells. (**C**) Quantification of invaded cells. Y-axis represents the relative number of cells (fold change) normalized to the corresponding negative control group. Experimental groups: mimic-NC: negative control for mimic; mimic-miR-7151-5p: cells overexpressing miR-7151-5p; inhibitor-NC: negative control for inhibitor; inhibitor-miR-7151-5p: cells with miR-7151-5p knockdown. One-way ANOVA with Tukey’s post hoc test was used for statistical comparisons: In (**B**): mimic-NC vs. mimic-miR-7151-5p: *p* < 0.01 (**), inhibitor-NC vs. inhibitor-miR-7151-5p: *p* < 0.0001 (****). In (**C**): mimic-NC vs. mimic-miR-7151-5p: *p* < 0.01 (**), inhibitor-NC vs. inhibitor-miR-7151-5p: *p* < 0.001 (***). Data are shown as the mean ± SD from three independent experiments. Magnification: 400×. Significance: *p* < 0.0001 (****), *p* < 0.001 (***), *p* < 0.01 (**).

Four experimental groups were included: miRNA-7151 overexpression group (mimic-miRNA-7151), mimic negative control (mimic-NC), miRNA-7151 inhibition group (inhibitor-miRNA-7151), and inhibitor negative control (inhibitor-NC). Following Transwell incubation, cells were visualized using crystal violet staining, visualized with a microscope at 400× magnification (Figure 4A), and subjected to quantitative analysis (Figure 4B,C).

The results indicated that miRNA-7151 overexpression significantly suppressed the migration and invasion capabilities of trophoblast cells. The mimic-miR-7151-5p group showed a significant decrease in the number of migrating and invading cells (*p* < 0.01 for both). In contrast, the inhibition of miRNA-7151 expression markedly enhanced HTR8 cell migration and invasion. In the inhibitor-miRNA-7151 group, the number of migrating cells increased significantly compared to the inhibitor-NC group (*p* < 0.001), along with a substantial rise in invading cells (*p* < 0.001).

### 3.5. RNA Extraction and Transcriptome Sequencing

**Figure 5:**
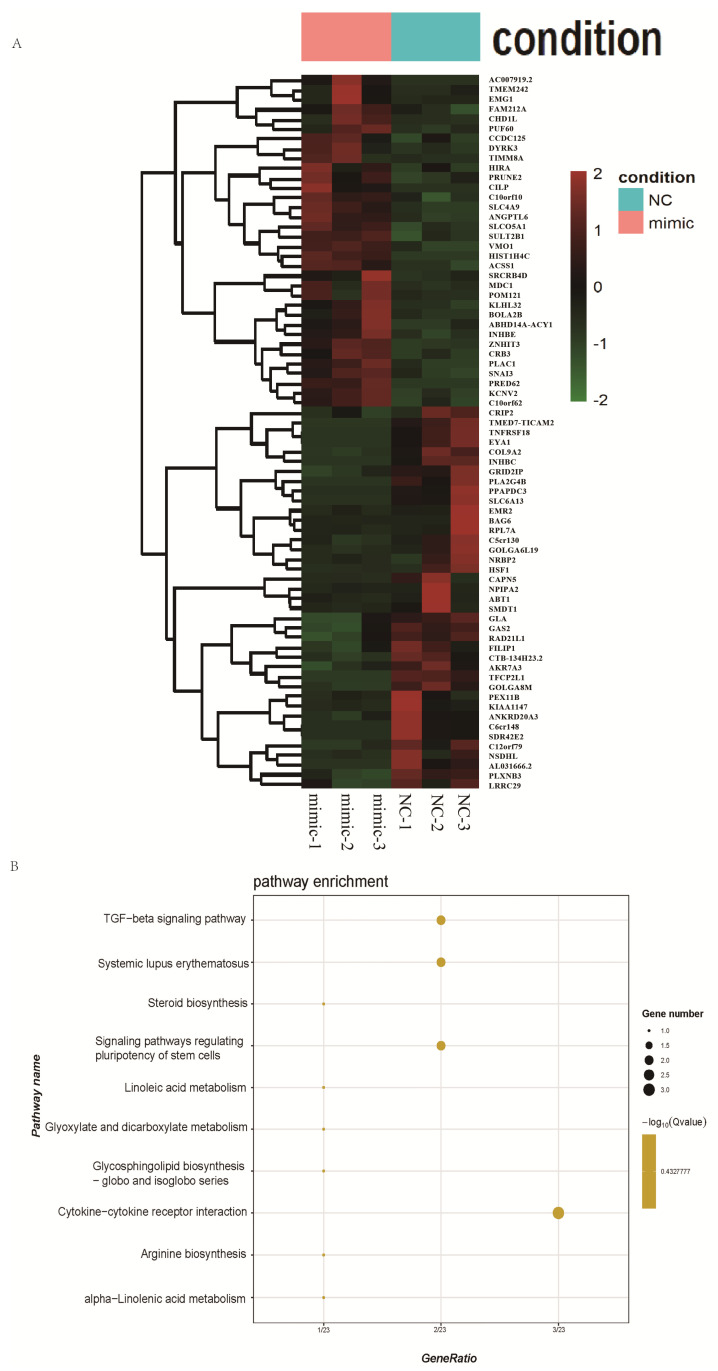
Transcriptomic and pathway analysis of HTR-8/SVneo cells transfected with miR-7151-5p mimic versus negative control (mimic_NC). To investigate the global transcriptomic changes induced by miR-7151-5p overexpression in trophoblast cells, RNA-seq was performed on HTR-8/SVneo cells transfected with either a miR-7151-5p mimic or mimic-NC. DEGs were identified and subjected to functional enrichment analysis, which was used to explore the underlying biological pathways potentially regulated by miR-7151-5p. (**A**) DEGs in the mimic_NC group, the thresholds for statistical significance were set at an adjusted *p* < 0.05 and |log_2_FC| ≥ 1. (**B**) KEGG pathway enrichment analysis for the mimic_NC group, pathways with adjusted *p* < 0.05 (Benjamini–Hochberg corrected), which were considered significantly enriched.

Comparative transcriptomic analysis revealed a distinct set of DEGs in the miRNA-7151 mimic group relative to the control. A heatmap of these genes (Figure 5A) showed clear differential gene expression patterns, with clustering analysis distinctly separating the mimic and NC samples. These findings indicate that miRNA-7151 overexpression induced a specific transcriptional response in trophoblast cells. Several genes related to transcriptional transduction, chromatin remodeling, and metabolic regulation were significantly up- or downregulated.

### 3.6. Gene Function Annotation and Enrichment Analysis

KEGG pathway enrichment analysis of DEGs between the mimic and mimic-NC groups showed significant enrichment in the TGF-β signaling pathway (Figure 5B). The TGF-β signaling pathway was essential for controlling cell proliferation and differentiation, migration, and immune modulation and has been reported to have key regulatory functions in the development of PE [45]. These results suggest that miRNA-7151 may influence trophoblast function via the TGF-β signaling pathway, thereby participating in PE pathogenesis.

Further DEG screening identified six genes in the mimic group closely associated with PE: *PLAC1*, *ANGPTL6*, *HIRA*, *GLA*, *HSF1*, and *BAG6* (Table 1). Among these, *PLAC1* (log_2_FC = 3.51), *ANGPTL6* (log_2_FC = 2.32), and *HIRA* (log_2_FC = 1.44) were significantly upregulated, while *GLA* (log_2_FC = −1.41), *HSF1* (log_2_FC = −1.92), and *BAG6* (log_2_FC = −4.51) were significantly downregulated (*p* < 0.05 for all). These genes are known to be involved in pregnancy-related pathological processes, placental development abnormalities, or maternal–fetal interface immune regulation [46,47,48,49,50,51,52], suggesting they may serve as key downstream effectors in miRNA-7151-mediated PE pathogenesis.

In summary, miRNA-7151 may participate in the pathological process of PE by regulating both the TGF-β signaling pathway and critical PE-associated genes.

### 3.7. PPI Analysis

**Figure 6:**
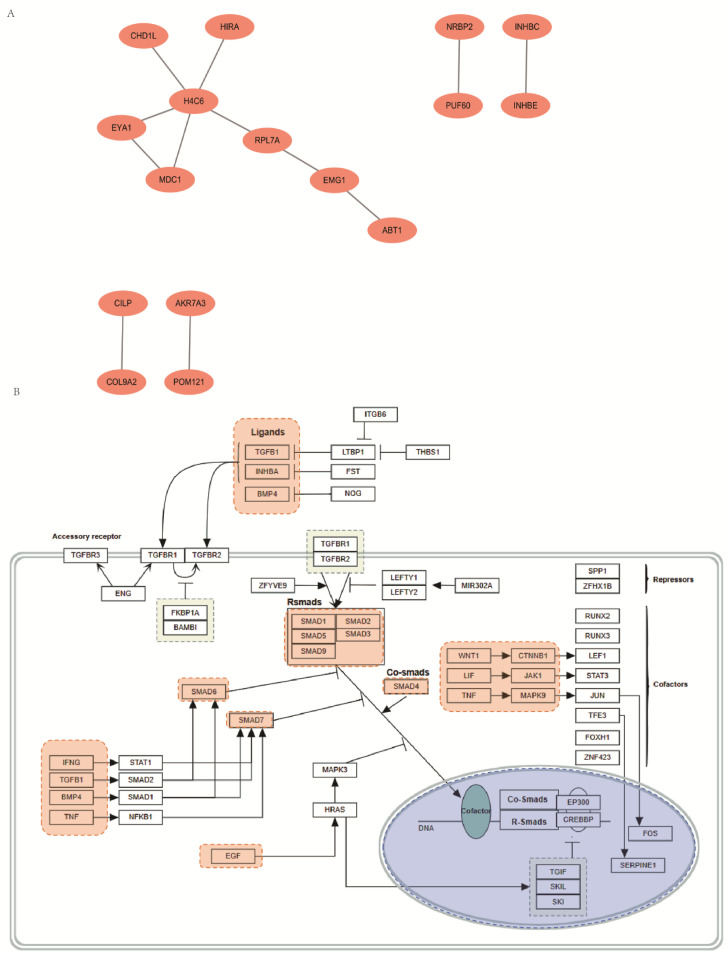
PPI network and TGF-β signaling pathway analysis of the miR-7151-5p mimic vs. NC group. To further elucidate the molecular mechanisms by which miR-7151-5p regulates trophoblast function, we analyzed the PPI networks and enriched signaling pathways based on the DEGs identified from transcriptome sequencing. (**A**) PPI network analysis of the mimic_NC group. (**B**) TGF-β receptor signaling pathway analysis.

Based on the RNA-seq results, a set of DEGs was used to construct a PPI network utilizing the STRING database (Figure 6A). The analysis showed that histone family members (such as *H4C6*) formed a highly interconnected core module within the network, suggesting that the chromatin structure and epigenetic regulation may play crucial roles in the biological processes modulated by miR-7151-5p. Additionally, genes such as *EMG1* and *CHD1L* were found to be connected—either directly or indirectly—with the core module, indicating their potential involvement in chromatin regulation-related processes.

### 3.8. Functional Enrichment Analysis

Further functional enrichment analysis confirmed that the TGF-β signaling pathway was significantly enriched among DEGs (Figure 6B). The pathway diagram revealed the involvement of several key components of this pathway, including *TGFβ1*, *BMP4* ligands, *TGFBR* receptors, and *SMAD* family members, all of which are central to TGF-β pathway activity. Downstream co-regulatory factors such as *FOS* and *FOXH1* were also differentially expressed, suggesting their participation in the transcriptional regulation of TGF-β-target genes [59]. Additionally, the presence of inhibitory regulators such as *SMAD6* and *SMAD7* suggests that the TGF-β pathway has a complex feedback regulation mechanism.

## 4. Discussion

Our study systematically illustrated that miR-7151-5p suppressed the migration and invasion of trophoblast cell by directly binding the lncRNA *KCNQ1OT1*, negatively regulating the Notch1 signaling pathway and affecting the TGF-β pathway. Our results uniquely demonstrate that miR-7151-5p modulates TGF-β signaling in trophoblast cells, potentially altering placental function in PE.

Using RNAhybrid prediction and the dual-luciferase reporter assays, we noticed that miR-7151-5p can target to the 3′UTR of *KCNQ1OT1*, which significantly suppresses its expression. These results aligns with previous studies; *miR-146a-3p* interacts with *KCNQ1OT1* to regulate trophoblast function via the CXCL12/CXCR4 pathway [19]. But the link between *miR-7151-5p* and *KCNQ1OT1* has not been previously reported. Our study is the first to identify this regulatory relationship, thereby filling an important gap in the understanding of lncRNA–miRNA interactions in PE pathogenesis.

Further analysis showed that the overexpression of miR-7151-5p not only suppresses *KCNQ1OT1* but also significantly reduces Notch1 mRNA levels. The Notch1 signaling pathway is linked to placental development and trophoblast cell function [60]. Its dysregulation contributes to the development of PE [2]. Our research demonstrates that miR-7151-5p might be associated with the pathogenesis of PE by modulating Notch1 signaling.

The results of the transwell assay illustrated that the overexpression of miR-7151-5p could markedly inhibit the invasion and migration of HTR8/SVneo trophoblast cells, whereas miR-7151-5p inhibition promotes these abilities. Our results aligned with previously published studies on miRNAs such as miR-210 and miR-362-3p, which also regulate trophoblast function in PE [13]. However, the specific mechanisms of miR-7151-5p in modulating trophoblast motility had not been previously characterized. Our study provides novel experimental evidence supporting its involvement in PE pathogenesis. Our evidence indicates that miRNA-7151 may play a key role in regulating signaling pathways related to cell motility, supporting the stability of the placental structure and influencing the invasion processes. It also provides an experimental basis for further research into its potential mechanisms in pregnancy-related disorders, such as preeclampsia and placenta accrete [61].

RNA sequencing and KEGG pathway analysis revealed that miR-7151-5p overexpression significantly impacts the TGF-β signaling pathway and regulates the expression of multiple PE-related genes, including *PLAC1*, *ANGPTL6*, *HIRA*, *GLA*, *HSF1*, and *BAG6* [46,47,48,49,50,51,52]. They are known to play important roles in placental development, immune regulation, and apoptosis, and their aberrant expression may contribute to the pathogenesis of PE [62]. By linking miR-7151-5p to both the TGF-β signaling pathway and a network of PE-related genes, this study expands our understanding of the molecular mechanisms through which miR-7151-5p may play a role in trophoblast dysfunction and the pathogenesis of PE.

Unlike previous research focusing on miRNAs such as miR-210 or miR-181, this research was the first to systematically explore the function and molecular mechanisms of miR-7151-5p in PE. We not only confirmed the direct interaction between miR-7151-5p and *KCNQ1OT1*, but also revealed that miR-7151-5p modulates trophoblast cell function through regulation of the Notch1 and TGF-β pathways, potentially contributing to PE pathogenesis. These findings suggest novel targets for the early screening and therapeutic intervention of PE.

## 5. Limitations

Although our study provides valuable insights, there are several limitations that need to be acknowledged. Firstly, all functional assays used the HTR-8/SVneo cell line, an immortalized first-trimester trophoblast model. While widely used, this in vitro system cannot fully recapitulate the complex architecture, heterogeneity, and maternal–fetal interactions present in the human placenta [63]. Therefore, the observed effects of miR-7151-5p on trophoblast migration and invasion should be interpreted with caution when extrapolating to in vivo placental development. Second, no primary placental tissues or clinical PE samples were included in this study. This limits the immediate translational applicability of our findings, and highlights the need for future validation in patient-derived placental tissues across different gestational stages and preeclampsia subtypes (e.g., early-onset vs. late-onset PE) [64]. Moreover, the study did not include the in vivo functional validation of miR-7151-5p’s role in placentation or PE pathogenesis. Future research should employ relevant rodent models of PE, such as transgenic and knockout mouse models that modulate miRNA expression in trophoblasts [65]. These systems would allow for the evaluation of the physiological consequences of miR-7151-5p dysregulation in vivo. Finally, although a transcriptomic analysis identified six DEGs as potential downstream effectors of miR-7151-5p, we did not perform qPCR or Western blot validation of these targets. Future studies should confirm the expression and functional roles of these DEGs to strengthen the mechanistic link between miR-7151-5p and PE pathogenesis.

**Figure 7:**
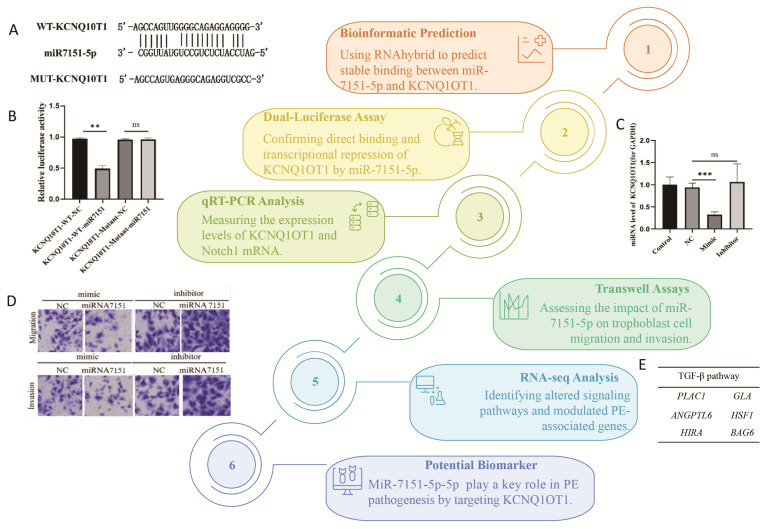
Workflow and key results of the study. An integrative approach combining RNAhybrid-based bioinformatic prediction (**A**), dual-luciferase reporter assays (**B**), qRT-PCR (**C**), transwell migration and invasion assays (**D**), and RNA-seq (**E**) was employed to systematically analyze the interaction between miR-7151-5p and *KCNQ1OT1* and assess their effects on trophoblast cell function behavior and gene expression. Significance: *p* < 0.001 (***), *p* < 0.01 (**), not significant (ns).

In conclusion, the workflow and key results of the study are shown in Figure 7. This study revealed the critical role of miR-7151-5p in the pathogenesis of PE. miR-7151-5p directly targets the lncRNA *KCNQ1OT1*, regulates the Notch1 signaling pathway, and affects trophoblast cell invasion and migration. Additionally, it modulates the expression of PE-related genes via the TGF-β signaling pathway, thereby contributing to the disease’s development. Together, these findings provide novel mechanistic insights and suggest that miR-7151-5p might be utilized as a predictive marker for the early screening of PE.

## Figures and Tables

**Table 1:** DEGs for miR-7151-5p mimic and NC group links with PE.

Ensembl_id	Gene	miR-7151-5p Mimic	NC	log2(FC)	*p*-Value
ENSG00000170965	*PLAC1*	0.121341831	0.009957439	3.50879365	0.012470628
ENSG00000130812	*ANGPTL6*	0.118435448	0.023556238	2.319557029	0.027106337
ENSG00000100084	*HIRA*	0.141205578	0.050841777	1.437466173	0.031709663
ENSG00000102393	*GLA*	0.897614745	2.397320911	−1.412718299	0.032110605
ENSG00000185122	*HSF1*	1.345940712	5.106967702	−1.91852461	0.009848118
ENSG00000204463	*BAG6*	0.025970633	0.596313102	−4.5121193	0.025031886

Note. DEGs between miR-7151-5p mimic and negative control (NC) groups related to preeclampsia (PE). Expression levels are shown as normalized values for each group. The log_2_ fold-change (log_2_FC) was calculated using the following formula: log_2_FC = log_2_(miR-7151-5p mimic expression/NC expression). The adjusted *p*-value < 0.05 and |log_2_FC| ≥ 1 were used to consider significantly differentially expressed genes. *p*-values were computed using the DESeq2 statistical model with Benjamini–Hochberg correction for multiple testing. Functional relevance summary (selected genes): PLAC1: placenta-specific gene linked with trophoblast proliferation and invasion; elevated in PE [53]. ANGPTL6: angiopoietin-like protein associated with endothelial function and PE-related vascular remodeling [54]. HIRA: a histone chaperone regulating trophoblast differentiation and embryogenesis [55]. GLA: deficiency may contribute to lysosomal dysfunction and oxidative stress in PE [56]. HSF1: key regulator of placental stress response; its dysregulation is implicated in PE [57]. BAG6: apoptosis regulator with roles in immune signaling and potential trophoblast viability [58].

## Data Availability

Raw data were generated at the Bio-X Institutes, Shanghai Jiao Tong University, and are available from the corresponding author upon request.
